# Reduced platelet count in mice protects against glucose intolerance and beta cell loss during a long-term high-fat diet

**DOI:** 10.3389/fimmu.2026.1854992

**Published:** 2026-07-01

**Authors:** Niklas Burkhard, Johannes Hoch, Shanshan Zhang, Muataz Ali Hamad, Nicolas Schommer, Carolin Mogler, Daniela Stallmann, Pierre Mangin, Krystin Krauel, Daniel Duerschmied, Nancy Schanze

**Affiliations:** 1 Cardiology and Angiology, Medical Center – University of Freiburg, Faculty of Medicine, University of Freiburg, Freiburg, Germany; 2 Cardiology, Haemostaseology, and Medical Intensive Care, Medical Centre Mannheim, Medical Faculty Mannheim, Heidelberg University, Mannheim, Germany; 3 European Center for AngioScience (ECAS), German Centre for Cardiovascular Research (DZHK) partner site Heidelberg/Mannheim, and Centre for Cardiovascular Acute Medicine Mannheim (ZKAM), Medical Centre Mannheim and Medical Faculty Mannheim, Heidelberg University, Mannheim, Germany; 4 Faculty of Biology, University of Freiburg, Freiburg, Germany; 5 Spemann Graduate School of Biology and Medicine (SGBM), University of Freiburg, Freiburg, Germany; 6 Department of Pediatrics, Harvard Medical School, Boston, MA, United States; 7 Institute of Pathology, School of Medicine and Health, Technical University Munich, Munich, Germany; 8 Université de Strasbourg, Institut National de la Santé et de la Recherche Médicale, Etablissement Français du Sang Grand-Est, Unité Mixte de Recherche-S1255, Fédération de Medecine Translationnelle de Strasbourg, Strasbourg, France; 9 Helmholtz-Institute for Translational AngioCardioScience (HI-TAC) of the Max Delbrück Center for Molecular Medicine in the Helmholtz Association (MDC) at Heidelberg University, Mannheim, Germany

**Keywords:** diabetes, glucose tolerance, insulin secretion, obesity, pancreatic beta cells, platelets, thrombocytopenia

## Abstract

**Introduction:**

Platelets promote hemostasis and modulate different inflammatory and immunological processes. Recently, it was reported that platelets interact with pancreatic beta cells to increase insulin secretion. Inflammatory conditions and a lack of sufficient insulin secretion drive the pathogenesis of type 2 diabetes (T2D) in the context of obesity. However, the specific role of platelets during the development of obesity-related T2D has not yet been investigated.

**Methods:**

Thrombopoietin receptor knockout (*MPL^-/-^*) mice displaying constant thrombocytopenia (approx. 90 % reduction in platelet count) or wild type (WT) mice were fed either a high-fat diet (HFD) or a control diet (CD) for 19 weeks, starting at 8 weeks of age. The mice were metabolically characterized at the end of the feeding period (metabolic caging, glucose and insulin tolerance testing, body and organ weight measurements, and gene expression analyses). Pancreatic tissue was histologically assessed via immunofluorescence, and circulating platelets were characterized by flow cytometry.

**Results:**

After HFD feeding, obese *MPL^-/-^* mice showed a significantly improved glucose tolerance compared to WT mice. At the same time, *MPL^-/-^* mice tended to exhibit a more pronounced insulin resistance. The weight gain of *MPL^-/-^*and WT mice did not differ significantly after 19 weeks of feeding, but body weight was higher in *MPL^-/-^* between weeks 4 and 12. Likewise, the weight of adipose tissue depots was similar between both genotypes at the end of the study. In metabolic cages, obese *MPL^-/-^* mice showed less physical activity than WT, while other parameters like O_2_-consumption and heat dissipation were similar between the two groups. The expression of genes involved in glucose metabolism in muscle, liver, and epididymal adipose tissue did not show relevant differences between *MPL^-/-^* and WT mice. At the end of the study, a significantly higher insulin concentration was measured in the circulation of obese *MPL^-/-^* mice. This was accompanied by a higher proportion of pancreatic beta cells in the total analyzed tissue area.

**Discussion:**

These results suggests that thrombocytopenia protects pancreatic beta cells in an obesity mouse model of T2D, thereby preserving insulin secretion and improving glucose tolerance.

## 1 Introduction

T2D is a global health burden with rising prevalence. In Germany, 8 million people suffered from this disease in 2023 (1). In the pathophysiology of T2D, the body’s insulin production is no longer sufficient for maintaining healthy glucose utilization (2). The development of this disease begins with a relative insulin deficiency. This is caused by inflammatory changes associated with obesity, which lead to peripheral tissues developing insulin resistance (3). Initially, insulin resistance can be compensated for by increased insulin secretion (2). However, in the long run, the increasing insulin demand together with other obesity-related triggers lead to greater beta cell stress and eventually beta cell death. Thereby, a reduced or absent insulin production of the pancreas further aggravates the disturbed glucose metabolism, and T2D becomes manifest (2). T2D drives micro and macrovascular secondary organ damage, giving rise to arteriosclerosis, myocardial infarction, and stroke (4). This illustrates the burden T2D puts on the healthcare system.

Platelets are anucleated cells that circulate for 8 to 12 days in human blood and approximately for 4 days in mouse blood before being eliminated by the liver and spleen. Platelet production is strictly regulated by thrombopoietin (TPO), which promotes platelet formation from megakaryocytes in the bone marrow (5). Beyond their classical role in hemostasis, platelets are involved in various inflammatory and immunological diseases, e.g., the chronic inflammation underlying atherosclerosis as well as the inflammatory response in ischemia-reperfusion injury following interventional therapy of myocardial infarction (6, 7). In obese humans, body mass index (BMI) is associated with platelet count, size, and reactivity (8–10). Additionally, elevated BMI has been linked to inflammatory platelet-derived transcripts, such as tumor necrosis factor (TNF), toll-like receptor (TLR) 2, and TLR4 (11). Given the inflammatory nature of the development of obesity-related T2D, a potential role for platelets seems well worth investigating.

Platelets contain serotonin, which is released upon activation (12). Notably, serotonin has been identified as a key factor in maintaining glucose tolerance in pregnant mice by stimulating beta cell proliferation (13). Furthermore, recent findings suggested that platelets can directly stimulate pancreatic insulin secretion (14). Under control diet conditions, mice lacking platelet receptors such as glycoprotein (GP) Ibα, GPVI, Gαq and Gα13, displayed impaired glucose tolerance compared to WT mice. Additionally, the lipid 20-Hydroxyeicosatetraenoic acid (20-HETE), released by platelets, was implicated in platelet-beta cell interaction (14).

Obesity-associated insulin resistance is accompanied by chronic low-grade inflammation and enhanced platelet activation (15, 16). Activated platelets release numerous inflammatory and bioactive mediators, including serotonin, cytokines, chemokines, and lipid metabolites, which may affect pancreatic islet biology (12–14). Since pancreatic beta cells are highly susceptible to inflammatory and metabolic stress, persistent platelet activation during obesity could contribute to beta cell dysfunction and exhaustion. However, despite emerging evidence for platelet–beta cell interactions, it remains unknown whether chronic alterations in platelet abundance influence beta cell adaptation and the progression toward T2D during obesity.

Therefore, this study aimed to investigate whether chronic thrombocytopenia alters glucose metabolism and beta cell adaptation during diet-induced obesity. To address this question, thrombopoietin receptor knockout (*MPL^-/-^*) mice with chronic thrombocytopenia were subjected to long-term high-fat diet feeding (17, 18).

## 2 Methods

### 2.1 Animals

Male *MPL^-/-^* mice on a C57BL/6J background were kindly provided by Pierre Mangin (INSERM, Strasbourg, France). Together with age-matched wild-type C57BL/6J *MPL^+/+^* mice, they were housed in the local animal facility (University Clinic Freiburg, Germany) at a 12 h light/dark cycle. Starting at an age of 8 weeks, the mice were fed either a high-fat diet (HFD, D12451, 45 kJ% fat, Ssniff-Spezialdiäten GmbH, Germany) or a control diet (CD, 3437 PXL15M/R, 4,5 kJ% fat, Granovit AG, Germany) for 19 weeks with *ad libitum* access to water and food. Body weight was determined weekly. Blood samples were collected from the tail vein every other week and were analyzed using the automated hematology analyzer Sysmex XN (Sysmex Corporation, Kobe, Japan). At the end of the study, mice were sacrificed with ketamine (100 mg/kg body weight) and xylazine (20 mg/kg body weight), followed by terminal cardiac blood collection. Male mice were used to reduce biological variability and because male C57BL/6J mice are known to develop a more robust obesity-associated metabolic phenotype under high-fat diet conditions compared to females (19). All experiments were conducted in accordance with the German animal protection law. All procedures had been approved by the federal authorities in Baden-Wuerttemberg, Germany (File # 35-9185.81/G-19/132).

### 2.2 Determination of food intake

Food consumption was determined either manually or using metabolic cages (Comprehensive Lab Animal Monitoring System, Columbus Instruments, see 2.3). For manual assessment, mice were placed in clean cages, and food consumption was calculated as the difference between the amount of food weighed in and the amount of food remaining within a 48 h period. A mean value was calculated for each cage based on the consumption of all mice housed together.

### 2.3 Metabolic caging

VO_2_ consumption, VCO_2_ production, respiratory exchange ratio (RER; V_CO2_/V_O2_), food intake, heat dissipation and locomotor activity for each of the analyzed mice were assessed for 48 h, using metabolic cages (Comprehensive Lab Animal Monitoring System, Columbus Instruments, USA; 24 h adaptation time, 24 h measurements).

### 2.4 Insulin and glucose tolerance testing

During weeks 17 and 18 of the feeding protocol, a glucose tolerance test (GTT) and an insulin tolerance test (ITT) were performed. For this purpose, mice were fasted for 12 h (GTT) or 2 h (ITT). Baseline blood glucose levels were determined via tail vein capping using a glucose meter (ACCU-CHECK Aviva, Roche Diabetes Care GmbH, Germany). Subsequently, 1 g glucose or 0.5 U human insulin per kg body weight was injected intraperitoneally (i.p.). Blood glucose levels were determined at 15, 30, 45, 60, 90, and 120 min post-injection, each value being determined in duplicates. Results were presented as the *area of the curve*, with the baseline subtracted at t=0 (20).

### 2.5 Analysis of mRNA expression

RNA was isolated using the RNeasy Lipid Tissue Mini Kit (Qiagen, Germany) according to the manufacturer’s instructions. RNA concentration and purity were determined using a Nanodrop spectrophotometer (ThermoFisher Scientific, USA). Subsequent cDNA synthesis was performed according to the manufacturer’s instructions using the iScript™ cDNA Synthesis Kit (Bio-Rad, USA). By using iQ™ SYBR^®^ Green Supermix (Bio-Rad, USA) and the gene-specific primer pairs (**Supplementary Table 1**), mRNA expression was measured and calculated using the 2^-ΔΔCt^ method normalized to hydroxymethylbilane synthase (HMBS) expression. Primer specificity was verified by gel electrophoresis.

### 2.6 Insulin concentration in serum

Insulin concentration was measured in the serum of the non-fasted mice at the end of the study. To obtain serum, whole blood was left at room temperature (RT) for at least 3 hours, then centrifuged (5 min, 1300 g, RT). The supernatant was collected and then stored at -80 °C until analysis. Insulin concentration in serum was analyzed using the Ultra-Sensitive Mouse Insulin ELISA Kit (Crystal Chem, USA) according to the manufacturer’s instructions.

### 2.7 Histology

Immediately after dissection, pancreas, epididymal white adipose tissue (epiWAT), and liver were fixed in phosphate-buffered 4% formaldehyde solution (Carl Roth, Germany) for 24 h. The fixed tissues were embedded in paraffin (Carl Roth, Germany), sectioned, and mounted onto microscope slides. Paraffin embedment as well as Hematoxylin and Eosin (HE) staining were performed by the Core Facility for Histopathology and Digital Pathology at the Medical Center – University of Freiburg according to standard protocols.

To quantify the proportion of pancreatic alpha and beta cells, immunofluorescence staining of the pancreatic tissue was performed. Firstly, deparaffinization and antigen unmasking were executed, followed by blocking non-specific antigens using normal donkey serum (Jackson ImmunoResearch, USA). Subsequently, the sections were incubated with the specific primary antibody or corresponding isotype control (**Supplementary Table 2**), followed by secondary antibody incubation (**Supplementary Table 3**). Finally, cell nuclei were stained with 4′,6-diamidino-2-phenylindole (DAPI, ROTI^®^Mount FluorCare DAPI, Carl Roth, Germany). Specific antibody binding was verified by isotype controls. The slices were imaged using the Axio Imager.Z2 (Carl Zeiss AG, Germany). For each mouse, two sections from different parts of the pancreas were analyzed. The analysis was based on a method described by Apaolaza et al. and conducted using the open-source software QuPath (21). Firstly, the total area of the analyzed tissue section was determined using DAPI cell nuclear staining. The islets of Langerhans were identified and classified accordingly. Endocrine cells within the islets were identified using the cell recognition function. Only islets containing at least 10 cells were included in the analysis. The classification into alpha and beta cells was based on the staining intensity for insulin and glucagon, respectively.

### 2.8 Sample preparation and flow cytometry analyses

20 µl of EDTA-anticoagulated blood were diluted with 180 µl Dulbecco’s phosphate-buffered saline containing Ca^2+^ and Mg^2+^ (DPBS, Gibco, ThermoFisher Scientific, USA). Subsequently, 90 µl of the resulting suspension was incubated in the dark with the flow cytometry antibodies (15 min, RT) (rat anti-mouse CD42b DyLight 649 (EMFRET, Germany), mouse anti-mouse/rat CD62P PE Cy7 (Biolegend, USA), rat anti-mouse CD41/61 PE (EMFRET, Germany)). Following staining, red blood cell lysis and fixation were performed (lysis/fix buffer, BD Biosciences, USA) (30 min, in the dark, RT). Samples were then centrifuged (5 min, 700 g, RT) and the resulting cell pellets were resuspended in 500 µl of DPBS. Samples were stored at 4 °C in the dark until analysis. Flow cytometry data was acquired using a FACS Canto II cytometer (BD, USA), with subsequent analysis using FlowJo version 10 (Tree Star, USA) (Gating strategy: **Supplementary Figure 1**).

### 2.9 Statistics and data presentation

Statistical analyses were based on predefined pairwise comparisons between WT and *MPL^-/-^* mice within the same dietary condition. As the study was not designed to investigate independent dietary effects or diet × genotype interactions, no statistical comparisons between dietary groups were performed. Statistical analysis was performed using GraphPad Prism 10 software (GraphPad Software, USA). Firstly, statistical outliers were identified and excluded using Grubb’s test. The Shapiro-Wilk test (confidence level: 95%) was then carried out to assess whether the data followed a Gaussian normal distribution. If a normal distribution was confirmed, an unpaired T-test was conducted; otherwise, a Mann-Whitney test was applied. When the unpaired T-test indicated significantly different standard deviations between groups, a Welch’s correction was applied accordingly. Glucose tolerance test (GTT) data were analyzed using a two-way repeated measures ANOVA followed by Sidak’s multiple comparisons test for *post hoc* analysis. Repeated measurements within individual animals were treated as matched values. P-values < 0.05 were considered statistically significant. Data were presented as mean ± SEM (standard error of the mean).

## 3 Results

### 3.1 MPL^-/-^ mice display preserved glucose tolerance despite comparable obesity during long-term HFD feeding

**Figure 1:**
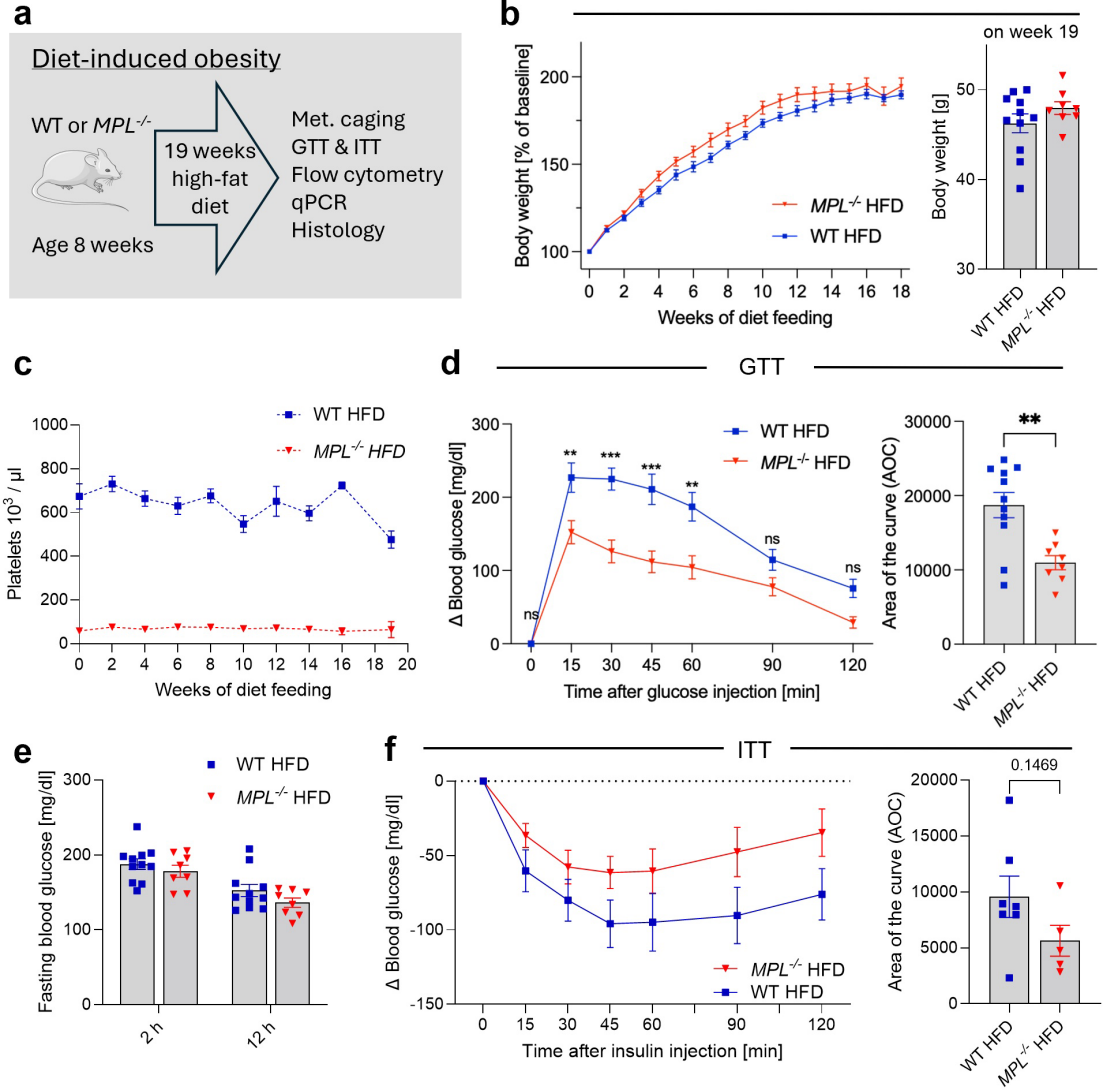
*MPL*-/- mice display preserved glucose tolerance during long-term HFD feeding. **(a)** Experimental set up of the high-fat diet (HFD) study. WT, wild type, *MPL^-/-^*, thrombopoietin receptor knockout, GTT, glucose tolerance testing, ITT, insulin tolerance testing, qPCR, quantitative polymerase chain reaction. **(b)** total body weight at the end of the study and body weight gain of male *MPL^-/-^* or WT mice fed a HFD **(c)** Platelet count of male *MPL^-/-^* or WT mice fed a HFD. **(d)** Glucose tolerance testing (GTT) conducted after 12 h fasting period along with the calculated area of the curve (AOC). **(e)** Fasting blood glucose after 2 or 12 hours of food deprivation. **(f)** Insulin tolerance testing (ITT) conducted after 2 h fasting period, along with the calculated area of the curve (AOC). **(b–e)** WT n=11, *MPL^-/-^* n=8; f, WT n=7, *MPL^-/-^* n=5; Results are presented as mean ± SEM. **p<0.01; ***p<0.001.

To investigate the role of platelets in obesity-associated glucose dysregulation, MPL^-/-^ and WT mice were fed a high-fat diet (HFD) for 19 weeks (**Figure 1a**). During HFD feeding, both genotypes continuously gained body weight and nearly doubled their initial weight over the course of the study. While final body weight did not differ significantly between groups, MPL^-/-^ mice transiently displayed higher body weights between weeks 4 and 12 of HFD feeding before weights converged at later stages (**Figure 1b**).

**Figure 2:**
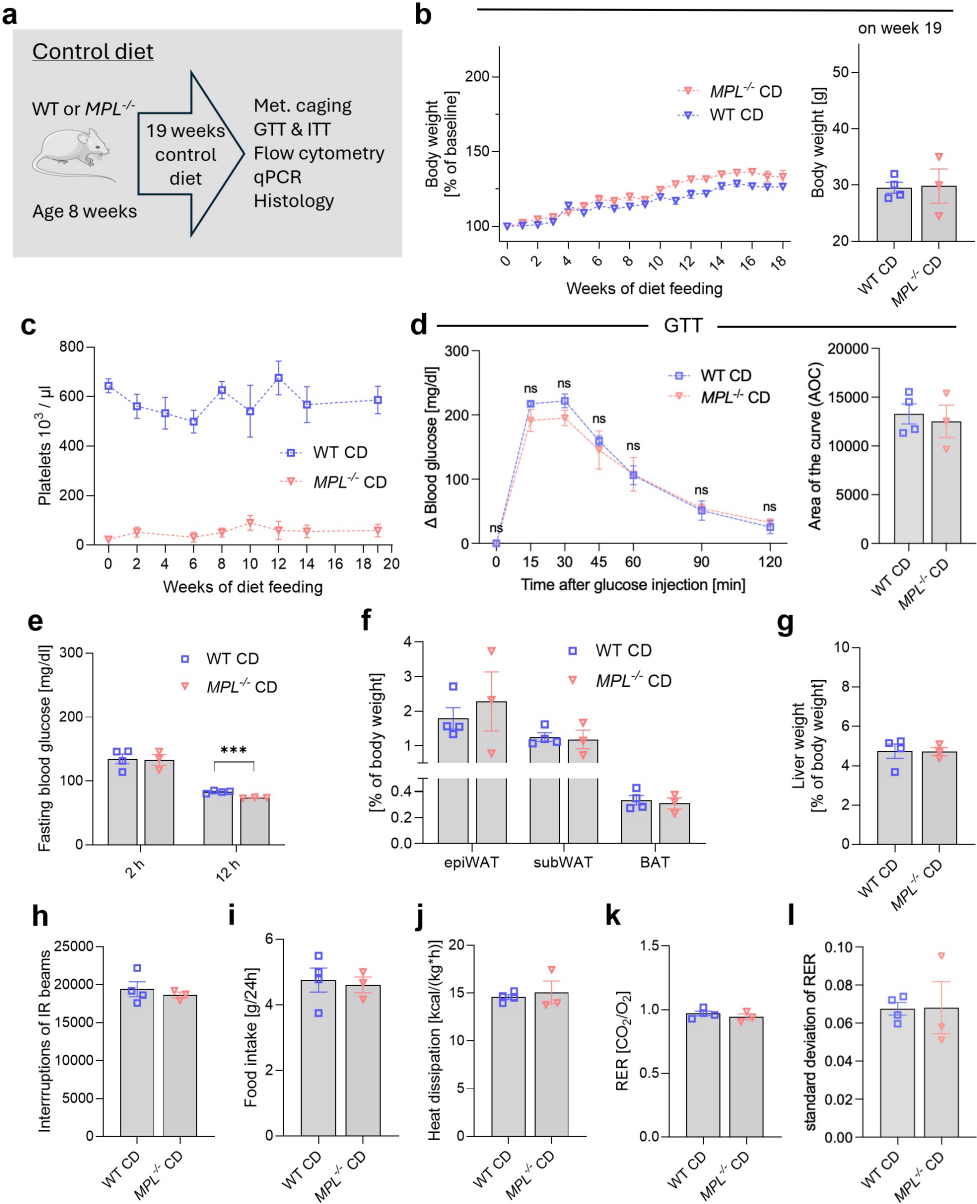
Basic metabolic characterization of *MPL^-/-^* and WT mice fed a control diet (CD). **(a)** Experimental setup of the control diet (CD) study. WT: wild type, *MPL^-/-^*: thrombopoietin receptor knockout, GTT: glucose tolerance testing, ITT: insulin tolerance testing, qPCR: quantitative polymerase chain reaction. **(b)** total body weight at the end of the study and body weight gain of male *MPL^-/-^* or WT mice fed a CD **(c)** Platelet count of male *MPL^-/-^* or WT mice fed a CD. **(d)** Glucose tolerance testing (GTT) conducted after 12 h fasting period along with the calculated area of the curve (AOC). **(e)** Fasting blood glucose after 2 or 12 hours of food deprivation. **(f)** Weights of epididymal white adipose tissue (epiWAT), subcutaneous white adipose tissue (subWAT), and brown adipose tissue (BAT) relative to body weight. **(g)** Liver weight relative to body weight. **(h–l)** Data obtained by metabolic caging analysis. **(h)** Physical activity of mice analyzed in metabolic cages. **(i)** Food intake of mice. **(j)** Heat dissipation. **(k)** Respiratory exchange ratio (RER). **(l)** Standard deviation of RER as a marker for its volatility. WT n=4, *MPL^-/-^* n=3. Results are presented as mean ± SEM. ***p<0.001.

As expected, MPL^-/-^ mice exhibited a pronounced chronic thrombocytopenia throughout the study period (**Figure 1c**). Despite the comparable obesity phenotype at the end of the intervention, obese MPL^-/-^ mice showed significantly improved glucose tolerance compared to WT mice after 17 weeks of HFD feeding (**Figure 1d**). In contrast, glucose tolerance did not differ between MPL^-/-^ and WT mice fed a control diet (CD) (**Figure 2d**). Fasting blood glucose levels after 2 h and 12 h fasting were comparable between HFD-fed groups (**Figure 1e**). Interestingly, obese MPL^-/-^ mice even tended to exhibit reduced insulin tolerance compared to WT mice (**Figure 1f**).

### 3.2 Improved glucose tolerance of obese MPL^-/-^ mice is not explained by altered energy balance or adiposity distribution

**Figure 3:**
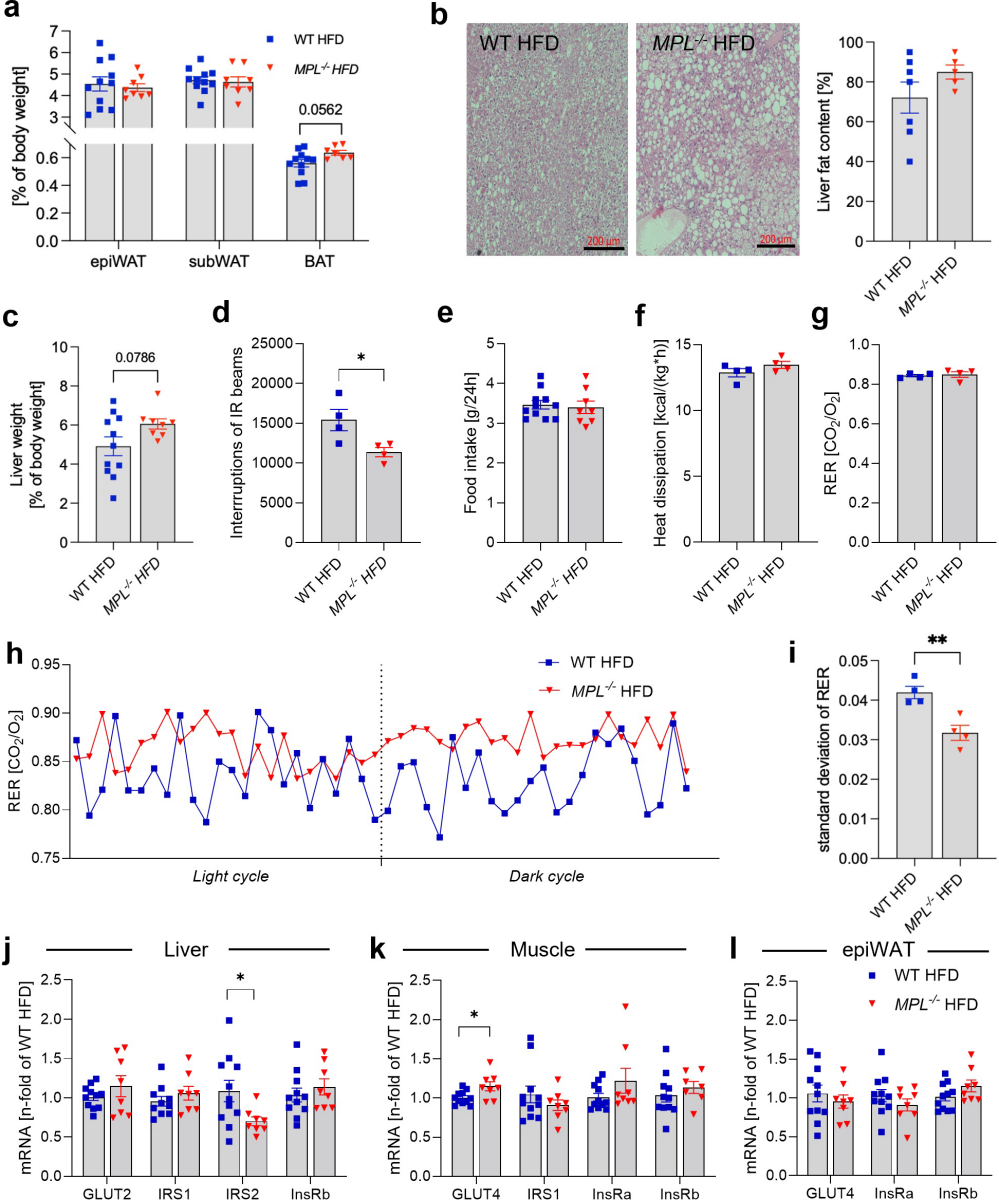
Energy and glucose metabolism of MPL^-/-^ and WT mice fed a high-fat diet (HFD). Male *MPL^-/-^* or WT mice were fed a high-fat diet (HFD) for 19 weeks starting at 8 weeks of age. **(a)** Weights of epididymal white adipose tissue (epiWAT), subcutaneous white adipose tissue (subWAT), and brown adipose tissue (BAT) relative to body weight. **(b)** Liver fat content and representative images of liver hematoxylin and eosin staining (HE). **(c)** Liver weight relative to body weight. **(d–i)**: Data obtained by metabolic caging analysis. **(d)** Physical activity of mice analyzed in metabolic cages. **(e)** Food intake of mice. **(f)** Heat dissipation. **(g)** Respiratory exchange ratio (RER). **(h)** RER over 24 h in one representative mouse of each genotype. **(i)** Standard deviation of RER as a marker for its volatility. **(j–l)**: Gene expression data obtained via qPCR. GLUT2/4: glucose transporter 2/4, IRS1/2: insulin receptor substrate 1/2, InsRa/b: Insulin receptor a/b. **(j)** Hepatic mRNA expression. **(k)** Skeletal muscle mRNA expression. **(l)** mRNA expression of epididymal white adipose tissue (epiWAT). **(a, c, e, j, k, l)** WT n=11, *MPL^-/-^* n=8. b: WT n=7, *MPL^-/-^* n=5. **(d, f, g, i)** WT n=4, *MPL^-/-^* n=4, h: one representative mouse per group. Results are presented as mean ± SEM. *p<0.05; **p<0.01.

Relative weights of epididymal and subcutaneous adipose tissue depots as well as additional relative organ weights were similar between genotypes after HFD feeding (**Figure 3a**; **Supplementary Figure 2**). Histological assessment suggested a tendency toward increased hepatic fat accumulation in MPL^-/-^ mice (**Figure 3b**), accompanied by a trend toward increased liver weight (**Figure 3c**). MPL^-/-^ mice also showed a tendency toward increased brown adipose tissue weight (**Figure 3a**).

To determine whether altered energy metabolism contributed to the preserved glucose tolerance of obese MPL^-/-^ mice, metabolic cage analyses were performed. Despite improved glucose tolerance, MPL^-/-^ mice showed reduced locomotor activity compared to WT mice (**Figure 3d**), while food intake remained unchanged (**Figure 3e**). In addition, heat dissipation and respiratory exchange ratio (RER) were similar between genotypes (**Figure 3f, g**). However, obese MPL^-/-^ mice displayed reduced RER variability over time, reflected by a lower standard deviation of RER values (**Figure 3h, i**), suggesting a more stable metabolic state.

Importantly, under CD conditions, body weight development, adipose tissue mass, liver weight, organ weights, physical activity, food intake, heat dissipation, and RER did not differ substantially between MPL^-/-^ and WT mice (**Figure 2**; **Supplementary Figure 2**).

### 3.3 Peripheral metabolic gene expression does not indicate markedly improved peripheral glucose utilization in obese MPL^-/-^ mice

To investigate whether improved peripheral glucose utilization contributed to the preserved glucose tolerance of obese MPL^-/-^ mice, expression of genes involved in glucose and insulin metabolism was analyzed in liver, skeletal muscle, and epididymal white adipose tissue.

In the liver, mRNA expression of Glucose transporter 2 (GLUT2), insulin receptor isoform b (InsRb), and insulin receptor substrate 1 (IRS1) did not differ between HFD-fed genotypes, whereas IRS2 expression was reduced in MPL^-/-^ mice (**Figure 3j**). In skeletal muscle, expression of IRS1, InsRa, and InsRb was similar between groups, while GLUT4 expression was slightly increased in obese MPL^-/-^ mice (**Figure 3k**). In epididymal adipose tissue, no relevant differences in GLUT4, InsRa, or InsRb expression were observed (**Figure 3l**).

Together with the tendency toward impaired insulin tolerance in MPL^-/-^ mice (**Figure 1f**), these findings do not indicate a consistent pattern of enhanced peripheral insulin sensitivity and suggest that improved glucose tolerance in obese MPL^-/-^ mice is unlikely to be primarily mediated by enhanced peripheral glucose utilization.

### 3.4 Obese MPL^-/-^ mice exhibit increased circulating insulin levels and preserved pancreatic beta cell area

**Figure 4:**
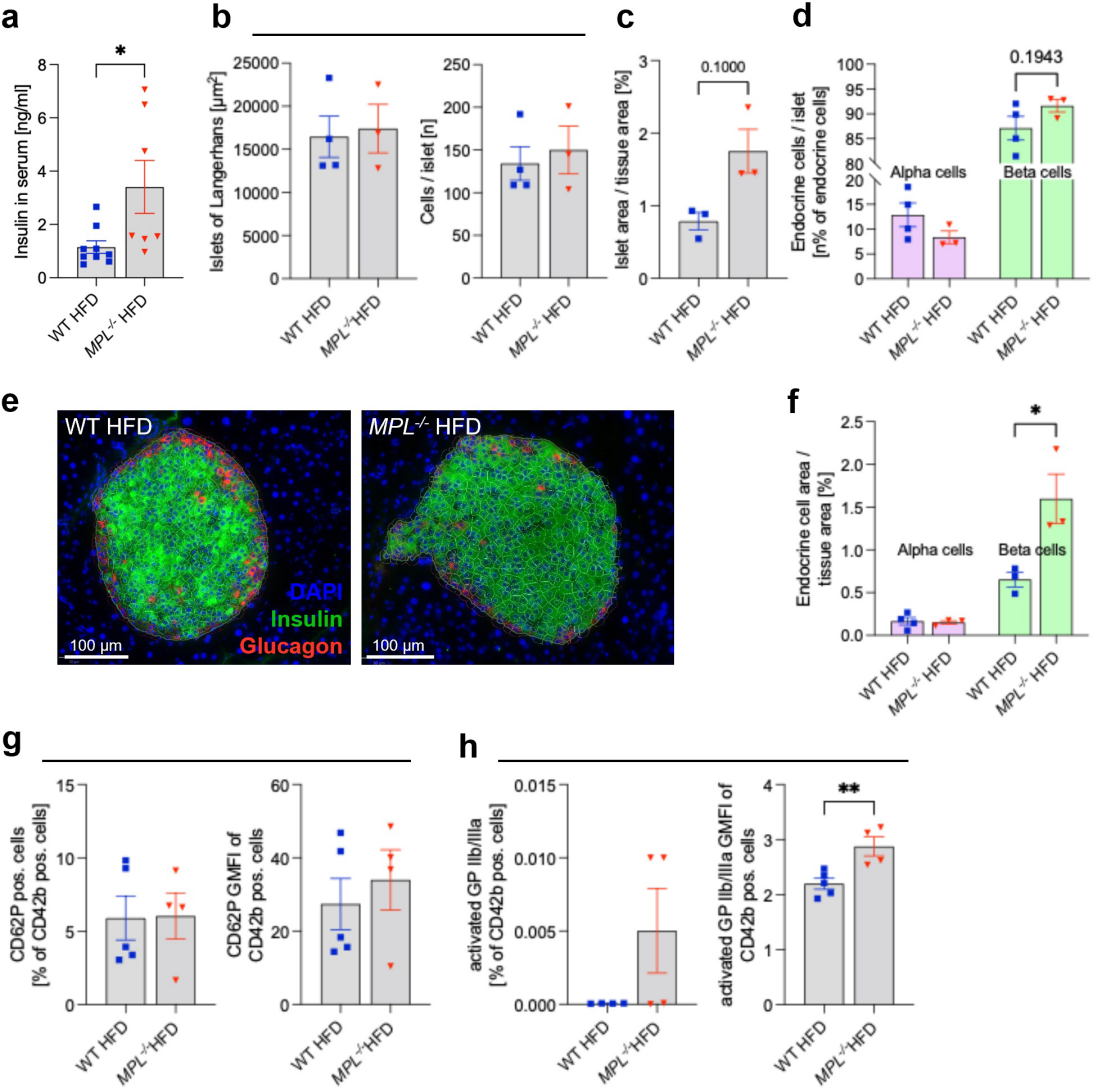
Pancreatic insulin metabolism and platelet characterization of *MPL^-/-^* and WT mice fed a high-fat diet (HFD). Male *MPL^-/-^* or WT mice were fed a high-fat diet (HFD) for 19 weeks starting at 8 weeks of age. **(a–h)** Data obtained from mice after chronic HFD feeding. **(a)** Randomly measured serum insulin levels. **(b–f)** Data obtained from immunofluorescence analysis of the pancreatic tissue. **(b)** Average islet area and average cell count per islet of Langerhans. **(c)** Proportion of islet area relative to total pancreatic tissue area. **(d)** Proportion of alpha and beta cells within an average islet of Langerhans. **(e)** Representative sections of the pancreatic tissue stained using immunofluorescence. **(f)** Proportion of endocrine cell area relative to total tissue area. **(g, h)** Flow cytometry analysis of blood. **(g)** Percentage of CD62P (P-selectin) positive cells and CD62P geometric mean fluorescence intensity (GMFI) of CD42b positive cells. **(h)** Activated GPIIb/IIIa positive cells and activated GPIIb/IIIa GMFI of CD42b positive cells. **(a)** WT n=9, *MPL^-/-^* n=7. **(b–f)** WT n=4, *MPL^-/-^* n=3. g, h: WT n=5, *MPL^-/-^* n=4. Results are presented as mean ± SEM. *p<0.05; **p<0.01.

To investigate whether preserved pancreatic function contributed to the improved glucose tolerance of obese MPL^-/-^ mice, serum insulin concentrations and pancreatic histology were analyzed at the end of the study. Obese MPL^-/-^ mice showed significantly higher circulating insulin levels compared to WT controls (**Figure 4a**).

Histological analysis of pancreatic tissue revealed no differences in average islet size or cell number per islet between genotypes (**Figure 4b**). However, MPL^-/-^ mice tended to exhibit a larger relative islet area and a higher proportion of beta cells within the islets (**Figure 4c–e**). Consequently, obese MPL^-/-^ mice showed a significantly greater proportion of insulin-producing beta cells relative to the total pancreatic tissue area (**Figure 4f**).

These findings suggest that chronic thrombocytopenia preserves pancreatic beta cell mass during obesity-associated metabolic stress, thereby supporting sustained insulin secretion and preserved glucose tolerance.

### 3.5 Residual platelets of MPL^-/-^ mice do not exhibit relevant preactivation

To determine whether the remaining platelets of MPL^-/-^ mice displayed altered activation characteristics, platelet preactivation markers were analyzed by flow cytometry. Expression of CD62P and activated GPIIb/IIIa was generally low in both genotypes. Neither the percentage nor the geometric mean fluorescence intensity (GMFI) of CD62P-positive platelets differed between groups (**Figure 4g**). Activated GPIIb/IIIa expression showed only minimal differences between MPL^-/-^ and WT mice (**Figure 4h**). Overall, residual platelets of obese MPL^-/-^ mice did not exhibit biologically relevant preactivation.

## 4 Discussion

We show here that chronic thrombocytopenia mitigates the deterioration of glucose metabolism during long-term HFD feeding, despite largely comparable obesity-associated metabolic alterations between MPL^-/-^ and WT mice. Interestingly, thrombocytopenic mice were transiently heavier between weeks 4 and 12 of HFD feeding (**Figure 1b**), suggesting that platelet count may contribute to early or intermediate phases of obesity development, whereas compensatory mechanisms may attenuate this association during prolonged HFD exposure. Improved glucose tolerance in obese MPL^-/-^ mice (**Figure 1d**) occurred in the absence of major differences in adiposity, food intake, or overall energy expenditure despite reduced locomotor activity (**Figure 3**), suggesting that the phenotype is unlikely to be primarily mediated by altered systemic metabolism or enhanced peripheral glucose utilization. Instead, our findings point toward a more direct platelet-dependent mechanism affecting pancreatic beta cell adaptation and insulin secretion during obesity-associated metabolic stress.

To further investigate the mechanisms underlying this phenotype, we examined major glucose-metabolizing organs and pancreatic insulin secretion more closely. Peripheral expression of genes involved in glucose and insulin metabolism showed no consistent pattern suggestive of markedly enhanced insulin sensitivity in MPL^-/-^ mice (**Figure 3**). MPL^-/-^ mice even showed a tendency toward impaired insulin tolerance, although this difference did not reach statistical significance (**Figure 1f**). Taken together, our data do not support substantially enhanced peripheral insulin sensitivity as the primary explanation for the improved glucose tolerance observed in obese MPL^-/-^ mice.

Nevertheless, we cannot exclude that alterations in peripheral metabolism contribute to the observed phenotype. Platelets have been shown to influence not only pancreatic beta cells but also peripheral metabolic tissues, including skeletal muscle (22). Therefore, it is conceivable that platelet-dependent effects on insulin-responsive tissues may have contributed to systemic metabolic adaptations, which in turn could promote compensatory beta-cell expansion and increased insulin secretion.

However, compensatory hyperinsulinemia alone is generally insufficient to fully preserve glucose tolerance in established long-term HFD models. Previous studies by Winzell and Ahrén as well as Sims et al. demonstrated that although insulin secretion initially increases to compensate for insulin resistance, prolonged HFD feeding progressively leads to impaired glucose tolerance despite persistent hyperinsulinemia (23, 24). Notably, Sims et al. showed that the initially compensated glucose tolerance phenotype was no longer maintained after 10 weeks of HFD feeding despite increased insulin secretion (24). Therefore, after 19 weeks of HFD feeding, the preserved glucose tolerance observed in MPL^-/-^ mice appears less likely to be explained solely by compensatory insulin hypersecretion secondary to peripheral metabolic alterations.

In contrast, obese MPL^-/-^ mice exhibited significantly higher circulating insulin levels together with a greater proportion of pancreatic beta cells relative to the total pancreatic tissue area (**Figure 4**), suggesting preserved pancreatic beta cell mass and sustained insulin secretion during obesity-associated metabolic stress.

There are several possible explanations for the preserved beta cell mass in MPL^-/-^ mice. Firstly, it has been reported that pancreatic beta-1 cells respond differently to hyperglycemia-induced stress depending on their genetic predisposition (25). Under stress, beta cells may either shift toward functional beta-4 cells that secrete insulin or toward dysfunctional beta-2/3 cells, which ultimately leads to beta cell death (25). It is conceivable that beta cells in MPL^-/-^ mice may possess a genetic advantage over WT mice. This possibility requires further investigation.

Secondly, reduced platelet-induced beta cell stress in MPL^-/-^ mice due to fewer platelet-beta cell interactions may have spared beta cells from exhaustion and death during the course of HFD feeding. Karwen et al. recently suggested that platelets directly influence insulin secretion from pancreatic beta cells (14). In their study, both genetic and pharmacological ablation of key platelet surface receptors (including GPIbα, GPVI, Gαq and Gα13) led to significantly impaired glucose tolerance due to reduced insulin secretion in mice. Furthermore, platelets were shown to release beta cell stimulating factors such as 20-HETE (14). Similarly, impaired platelet dense granule release due to Unc13d deficiency was associated with reduced insulin secretion and impaired glucose tolerance (26).

Importantly, our study differs fundamentally from the work by Karwen et al., who investigated platelet–beta cell interactions by disrupting key platelet signaling pathways, including GPIbα, GPVI, Gαq, and Gα13 (14). While complete disruption of platelet signaling impairs insulin secretion and glucose tolerance, our findings in a long-term HFD suggest that platelet effects on pancreatic beta cells may be context dependent. Therefore, while physiological platelet signaling seems to be required for basal insulin secretion, we hypothesize that excessive platelet-mediated stimulation during chronic obesity may contribute to beta cell exhaustion and dysfunction.

In contrast to GPIbα-, GPVI-, Gαq-, Gα13-, and Unc13d-deficient mice, in which platelet function is impaired in all circulating platelets (14, 26), MPL^-/-^ mice retain approximately 10% residual circulating fully functional platelets, which may still be sufficient to preserve basal platelet-mediated beta cell stimulation and insulin secretion under physiological conditions. Upon activation, platelets increase the expression of surface molecules and the release of granula (27, 28). Given that platelet activation is elevated in both humans and mice suffering from hyperglycemia (14, 29), one possible explanation is that increased blood glucose in our WT HFD model promoted excessive platelet-beta cell interactions. This may initially enhance insulin secretion but subsequently contribute to beta cell stress during prolonged HFD feeding. In MPL^-/-^ on the other hand, the markedly reduced platelet count may have limited such excessive platelet-mediated beta cell stimulation during chronic metabolic stress allowing beta cells more time to adapt to the increased insulin demand during prolonged HFD feeding. This may have protected beta cells from exhaustion and cell death and, by preserving beta cell integrity, may also have enabled subsequent beta cell expansion and sustained insulin secretion over time. A similar adaptive mechanism has previously been described in a murine model of chronic hyperglycemia, in which beta cells transiently downregulated the glucose sensor GLUT2 in order to reduce glucose-induced stress, thereby allowing cellular adaptation, preventing beta cell death, and ultimately preserving long-term beta cell function (25).

To rule out the possibility that the remaining platelets in MPL^-/-^ mice are pre-activated due to the knockout or exhibit altered behavior, we examined them using flow cytometry. No biologically relevant differences in platelet preactivation were measured in the blood of obese MPL^-/-^ mice (**Figure 4g, h**). Additionally, previous studies have described platelets of MPL^-/-^ mice as morphologically and functionally normal (17, 30). Furthermore, the impact of the whole-body MPL^-/-^ knockout on other cell types must also be considered, as previous studies have reported a in general reduced number of hematopoietic progenitor cells in these mice (17). However, despite these developmental changes, mature peripheral blood cell counts were largely preserved in earlier MPL-deficient models (31, 32). Consistent with these observations, we did not detect relevant differences in circulating leukocyte counts or erythrocyte counts between MPL^-/-^ and WT mice at the end of the study (**Supplementary Figure 3**). Therefore, the observed metabolic phenotype is unlikely to be explained by major alterations in mature non-platelet hematopoietic cell populations. Taken together, this supports our hypothesis of limited platelet-induced beta cell stress in obese MPL^-/-^ mice.

From a translational perspective, our findings raise the possibility that platelet-driven inflammatory or secretory pathways contribute to progressive beta cell dysfunction during obesity. Although therapeutic thrombocytopenia is clearly not a clinically feasible strategy, selective modulation of platelet activation or platelet-derived mediators could represent a potential future approach to reduce beta cell stress and preserve pancreatic function in patients at risk for T2D.

Some limitations of the work should be mentioned. Firstly, we performed predominantly single-point measurements at the end of the feeding period. Future studies with repeated measurements throughout the feeding regimen would help to clarify the temporal sequence of metabolic and pancreatic alterations. Secondly, several analyses were performed with relatively small sample sizes, particularly histological and metabolic cage experiments, which may have limited statistical power for subtle phenotypic differences. Thirdly, although our data support a role of preserved beta cell function in the improved glucose tolerance of MPL^-/-^ mice, the precise contribution of peripheral insulin signaling remains incompletely understood. While expression of key genes involved in glucose and insulin metabolism did not reveal major differences between genotypes and insulin tolerance was not significantly altered, future studies should assess insulin signaling at the protein level, including phosphorylation of key signaling molecules in insulin-responsive tissues under basal conditions and following insulin stimulation. Furthermore, our findings should be validated in female mice, as sex differences are known to influence susceptibility to HFD-induced metabolic syndrome (19). Finally, the findings were obtained in a murine model of diet-induced obesity and therefore require validation in human studies before translational conclusions can be drawn.

Taken together, our findings identify platelets as potential modulators of pancreatic beta cell adaptation during obesity-associated metabolic stress. In contrast to previous studies focusing on physiological platelet support of insulin secretion, our data suggest that chronic platelet reduction may protect beta cells from exhaustion during prolonged HFD feeding. These findings broaden the current understanding of platelet functions in metabolic disease and suggest that selective modulation of platelet-derived signaling pathways may represent a future strategy for preserving beta cell function in obesity-associated T2D.

## Data Availability

The raw data supporting the conclusions of this article will be made available by the authors, without undue reservation.
