# Supplementary material for: Reduced platelet count in mice protects against glucose intolerance and beta cell loss during a long-term high-fat diet
Source: Front Immunol. 2026 Jul 1;17:1854992. doi: 10.3389/fimmu.2026.1854992 (PMC13368534; doi:10.3389/fimmu.2026.1854992)
Supplement: Supplementary file 1 [file DataSheet1.pdf]

Supplementary Material

Reduced platelet count in mice protects against glucose intolerance and beta cell loss during a long-term high-fat diet

Niklas Burkhard[1], Johannes Hoch[1], Shanshan Zhang[1,2], Muataz Ali Hamad[1,3,4], Nicolas Schommer[1,2,5], Carolin Mogler[6], Daniela Stallmann[1], Pierre Mangin[7], Krystin Krauel[2,5,8], Daniel Duerschmied[2,5,8*], Nancy Schanze[1,2,5,8]

*** Correspondence:** Daniel Duerschmied, Daniel.duerschmied@umm.de

1 Supplementary Figures

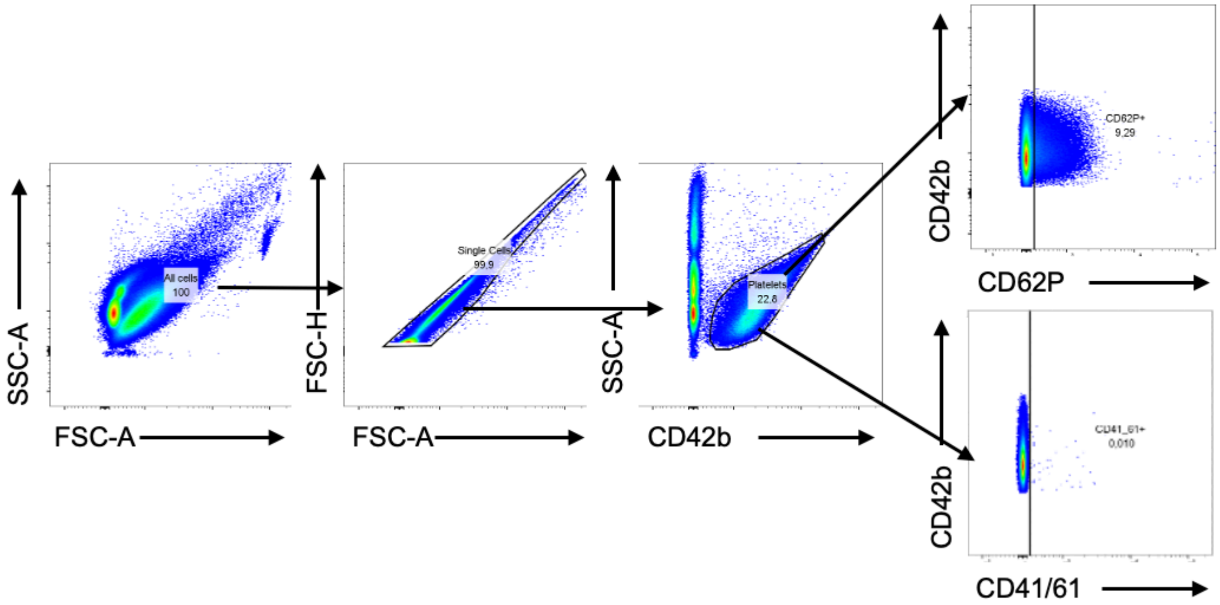

Supplementary Figure S1: Gating strategy of flow cytometry analysis. Flow cytometry was used to assess platelet pre-activation by measuring CD62P (P-selectin) and activated GPIIb/IIIa (CD41/61) expression. For this purpose, doublets (FSC-H, FSC-A) were first excluded. The platelet population was subsequently narrowed down (CD42b) and plotted against CD62P and CD41/61.

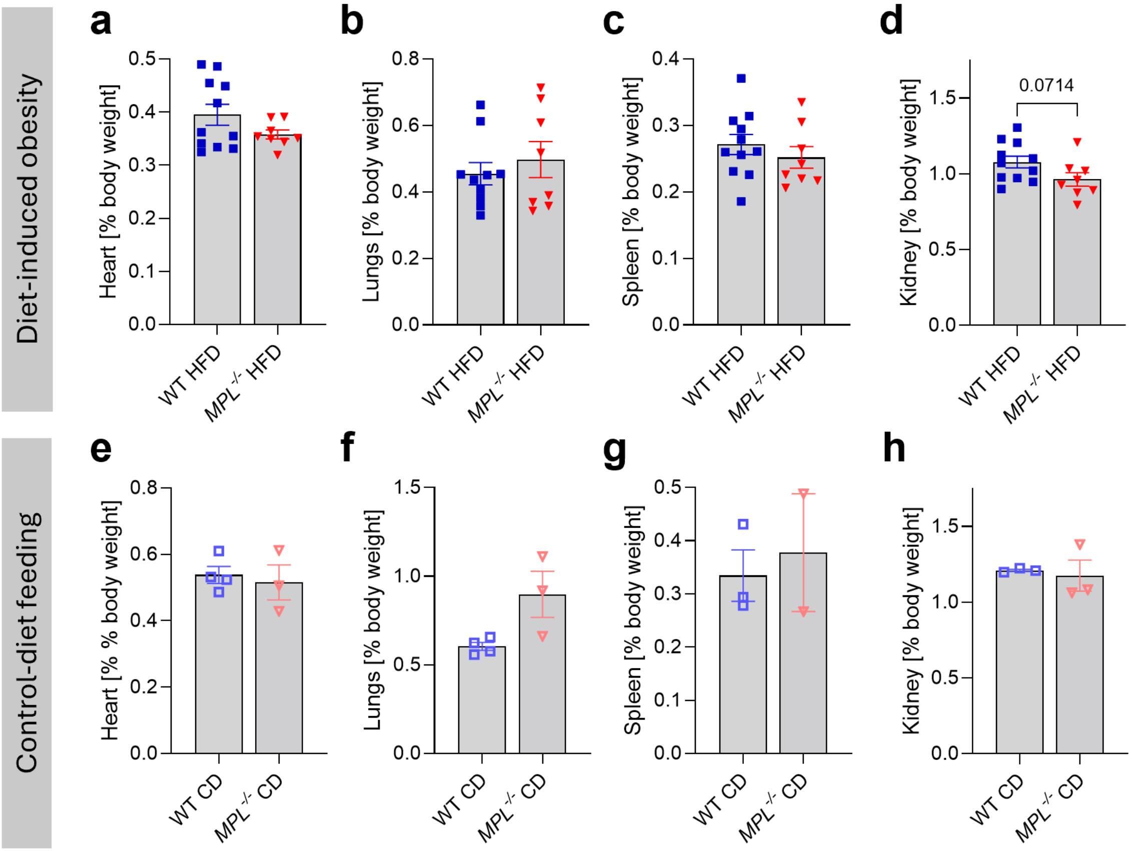

Supplementary Figure S2: Additional data of mice fed a high-fat diet (HFD) or control diet (CD). a – d: Heart, lung, spleen, and kidney weights (% of body weight) obtained from mice after chronic HFD feeding. e – h: Same parameters obtained from mice after CD feeding. a - d: WT HFD n=11, *MPL*$^{-/-}$ HFD n=8. e - h: WT CD n=4, *MPL*$^{-/-}$ CD n=3. Results are presented as mean±SEM.

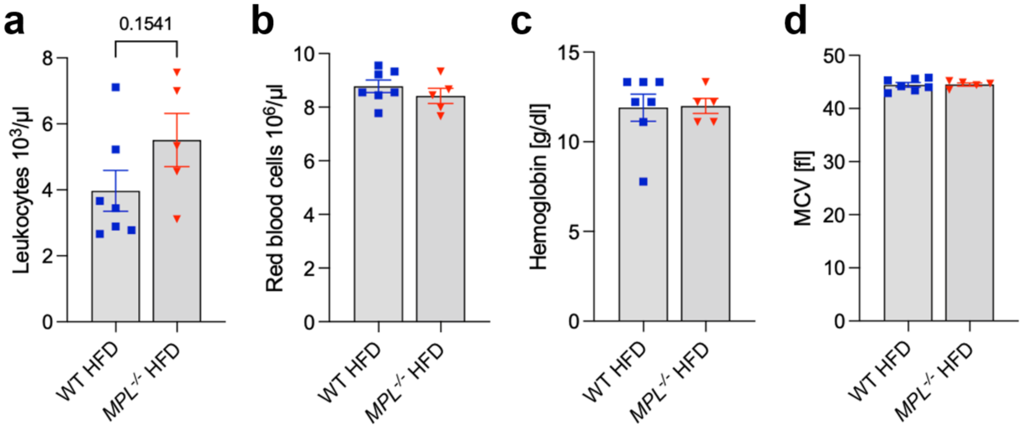

Supplementary Figure S3: Blood analysis of HFD-fed mice at the end of the study. a: white blood cell count. b: red blood cell count. c: hemoglobin. d: mean corpuscular volume (MCV). WT HFD n=7, *MPL*$^{-/-}$ HFD n=5. Results are presented as mean±SEM.

2 Supplementary Tables

Supplementary Table S1: Sequences of gene specific oligonucleotides used for qPCR analyses

Gene	forward 5’-3’	reverse 5’-3’	reference
Hydroxymethyl bilane synthase (HMBS)	ATGAGGGTGATTCGA GTGGG	TTGTCTCCCGTGGTGGAC ATA	(1)
Insulin receptor substrate 1 (IRS1)	CGATGGCTTCTCAGAC GTG	CAGCCCGCTTGTTGATGTT G	(2)
Insulin receptor substrate 2 (IRS 2)	CTGCGTCCTCTCCCAA AGTG	GGGGTCATGGGCATGTAG C	(2)
Glucose transporter type 4 /SLC2A4 (GLUT4)	GTGACTGGAACACTG GTCCTA	CCAGCCACGTTGCATTGT AG	(2)
Glucose transporter type 2/SLC2A2 (GLUT2)	TCAGAAGACAAGATC ACCGGA	GCTGGTGTGACTGTAAGT GGG	(2)
Insulin receptor A (InsRa)	TCCTGAAGGAGCTGG AGGAGT	CTTTCGGGATGGCCTGG	(3)
Insulin receptor B (InsRb)	TCCTGAAGGAGCTGG AGGAGT	TTCGGGATGGCCTACTGT C	(3)

Supplementary Table S2: Primary antibodies and isotype controls for immunofluorescence staining of pancreas tissue

Antibody	Dilution	Manufacturer
Insulin - 20-IP30	1:250	Fitzgerald Industries – Biosynth AG, Staad, Swiss
Glucagon - 2760S	1:100	Cell Signaling Technology, Danvers (MA), USA
Insulin Isotype - guinea pig - ABIN5633231	1:250	Antibodies-online.com, Limerick (PA), USA
Glucagon Isotype – rabbit - #2729	1:10.000	Cell Signaling Technology, Danvers (MA), USA

Supplementary Table S3: Secondary antibodies for immunofluorescence staining of pancreas tissue

Antibody	Dilution	Manufacturer
guinea pig IgG-Cy2 - 706-225-148	1:500	Jackson ImmunoResearch, West Grove (PA), USA
rabbit IgG-Cy3 - 711-165-152	1:200	Jackson ImmunoResearch, West Grove (PA), USA

3 Supplementary References

1. Zhang J, Tang H, Zhang Y, Deng R, Shao L, Liu Y, et al. Identification of suitable reference genes for quantitative RT-PCR during 3T3-L1 adipocyte differentiation. Int J Mol Med. 2014;33(5):1209-18.

2. https://pga.mgh.harvard.edu/primerbank/

3. https://faseb.onlinelibrary.wiley.com/doi/full/10.1096/fj.202100497RR
